# Pharmacokinetic profile of phenytoin in dried blood spot with high-performance liquid chromatography—photodiode array

**DOI:** 10.3389/fphar.2024.1326996

**Published:** 2024-06-26

**Authors:** Yahdiana Harahap, Limeylia Ng, Sunarsih Sunarsih

**Affiliations:** ^1^ Bioavailability/Bioequivalence Laboratory, Faculty of Pharmacy, Universitas Indonesia, Depok, Indonesia; ^2^ Faculty of Military Pharmacy, Republic of Indonesia Defense University, Bogor, Indonesia; ^3^ Dea Medika Clinic, Bogor, Indonesia

**Keywords:** phenytoin, carbamazepine, dried blood spot, high-performance liquid chromatography -photodiode array, epilepsy

## Abstract

Phenytoin is a first-line antiepileptic drug with narrow therapeutic range and follows non-linear pharmacokinetics. Pharmacokinetics of phenytoin have been studied in plasma matrix before, however, there were several disadvantages. This study aimed to obtain partial validation data of the analytical method and the pharmacokinetic profile of phenytoin in Dried Blood Spot (DBS) of six healthy subjects. DBS has the advantage of only requiring small sample volumes and could be transported more efficiently. Phenytoin along with carbamazepine as the chosen internal standard was analyzed with a reversed-phase high performance-liquid chromatography system and a photodiode array detector at 205 nm. The results of partial validation, which evaluated the linearity, within-run accuracy, and precision, were within the criteria acceptance range. The pharmacokinetic profile showed that average AUC_0-t_ was 83.81 ± 37.32 μg.h/mL and AUC_0-∞_ was 83.65 ± 38.89 μg.h/mL with an average ratio of 93%. Previous study quantifying phenytoin in the plasma matrix found the average AUC_0-t_ was 39.41 ± 8.57 µg.h/mL and AUC_0-∞_ was 42.94 ± 9.55 µg.h/mL. Despite the difference between parameters of phenytoin analyzed in DBS and plasma matrices, the pharmacokinetic profiles obtained from both matrices were similar indicated by comparable concentration-time curves, thus, proving that DBS matrix can be used interchangeably with the plasma matrix as a more comfortable and effective alternative to phenytoin quantification in blood.

## 1 Introduction

Phenytoin is the first-line antiepileptic drug for tonic-clonic seizures and focal seizures acting through blockade of voltage-gated sodium channel (Qiao et al., 2014). During administration of phenytoin therapy, total drug concentration needs to be in the range of 10–20 μg/mL. However, Singu et al. (2022) reported that the phenytoin concentration in 41.1% of patients did not reach the minimum effective concentration and 12.5% of patients exceeded the minimum toxic concentration (20 μg/mL). In most patients (57.1%), the concentration of unbound phenytoin was above the appropriate therapeutic range of 1–2 μg/mL.

The problems encountered in phenytoin therapy can be attributed to the characteristics of the drug. Phenytoin has great interindividual pharmacological variability and a narrow therapeutic index (Sumarno et al., 2023). In the distribution phase, more than 90% of phenytoin is bound to protein and does not produce a pharmacological response (Alexander et al., 2018). Small changes in plasma protein-bound phenytoin concentrations can significantly affect plasma free phenytoin concentrations, for example, due to the presence of other drugs that inhibit protein binding of phenytoin in plasma (Alexander et al., 2018). In addition, phenytoin also follows nonlinear pharmacokinetics (Sumarno et al., 2023). If the metabolic processes of phenytoin are saturated, small dose increases can produce large increases in plasma phenytoin concentrations and trigger toxic effects. Therefore, frequent monitoring of phenytoin concentrations during therapy is necessary to ensure the safety and efficacy of therapy.

A study of the pharmacokinetics of phenytoin in the plasma matrix in healthy subjects has been carried out previously by Rojanasthien et al. (2007) who used HPLC-UV. Dalmora et al. (2009) have also conducted a study of phenytoin pharmacokinetics using HPLC-PDA. However, the blood sampling technique used in both studies were invasive and required high sample volumes, causing patient discomfort (Sathyanarayanan and Somashekara, 2022). Extraction of plasma samples required large amounts of organic solvent and produced large amounts of waste (Hemmati et al., 2020).

Dried Blood Spot (DBS) matrix is an alternative to plasma matrix. DBS only requires a small volume of blood sample which can be taken using the fingerprick technique to facilitate the blood sampling procedure (Sathyanarayanan and Somashekara, 2022). Patient comfort is also increased because blood samples are taken in a minimally invasive manner. The dried DBS samples remained stable at room temperature. The sample handling and transportation process is also more cost-effective and time-effective because the sample does not need to be centrifuged beforehand, does not need to be extracted with large amounts of organic solutions, and does not need to be shipped under specific storage conditions, such as using a refrigerator and dry ice (Xu et al., 2012). Hence, pharmacokinetic profile of phenytoin in DBS of healthy subjects needed to be obtained to determine if DBS matrix can be used interchangeably with plasma.

## 2 Materials and methods

### 2.1 Chemicals and reagents

Phenytoin (BPFI, Indonesia), carbamazepine as internal standard (BPFI, Indonesia), ultrapure water grade (Satorius Walter Filter), acetonitrile HPLC grade, methanol HPLC grade, Dried Blood Spot paper (Whatman 903, United States), and a matrix in the form of whole blood (Indonesian Red Cross).

### 2.2 High-performance liquid chromatography—photodiode array

The HPLC-PDA system consisted of a LC–20AD pump (Shimadzu, Japan); an autosampler SIL-20A (Shimadzu, Japan); a C_18_ SunFire™ column (5 μm; 4.6 mm × 250 mm) (Waters, United States); a Waters 2966 photodiode array detector (Waters, United States of America); and Empower 2 Chromatography Data Software (Waters, United States of America) installed in a computer with a central processing unit (Dell, United States of America). Analysis was performed using a combination of methanol, acetonitrile and water (44:10:46, v/v/v) as the mobile phase; flow rate was 1.0 mL/min; column temperature was 35°C; with a photodiode array detector at a wavelength of 205 nm, which followed the analytical method developed and fully validated by Jihan (2023) from the same laboratory.

### 2.3 Sample for analysis

The sample used was dried blood spots prepared from whole blood samples of 6 healthy subjects, who had taken conventional 100 mg phenytoin capsules (PT. Ikapharmindo Putramas), using the finger prick technique. Sample size was below the required number of subjects for pharmacokinetics study, which was 12 subjects according to national guidelines (Tata Laksana Uji Bioekivalensi, 2022), due to limited funding. The subject’s inclusion criteria were healthy subjects aged 18–55 years; a body mass index of 18.5–24.9 kg/m^2^; subjects with normal physical examination results and vital signs; subjects who were not taking other drugs 14 days before and during the study; non-smoking subjects; subjects with no history of alcohol dependence or use of illicit drugs; subjects who were willing not to consume food and drinks that could affect the function of the digestive tract, circulation, liver and/or kidneys, such as tea, coffee, fruit juices and soft drinks for 24 h before blood collection; and subjects who have stated their willingness to participate in the research and signed the informed consent sheet. Subjects were excluded if they were diagnosed positive for COVID-19; had a history of cardiovascular, kidney, liver, lung, gastrointestinal, endocrine, nervous, and hematological diseases; and contraindicated or hypersensitive to phenytoin.

### 2.4 Preparation of stock and working solutions

A 1,000 μg/mL stock solution was prepared by dissolving 10.0 mg standard phenytoin in 10.0 mL methanol in a volumetric flask. Then, the solution was sonicated for 10 min. The 1,000 μg/mL phenytoin stock solution was diluted with methanol to obtain a working standard solution with a concentration of 1 μg/mL; 10 μg/mL; 50 μg/mL; 100 μg/mL; 200 μg/mL; 300 μg/mL; and 500 μg/mL.

### 2.5 Preparation of calibrators and quality control samples

The working standard solution was diluted with blood to obtain a calibration curve standard solution with a concentration of 0.1 μg/mL; 1 μg/mL; 5 μg/mL; 10 μg/mL; 20 μg/mL; 30 μg/mL; and 50 μg/mL. The working standard solution of phenytoin was diluted with methanol to obtain a quality control solution with three concentrations, which were 0.3 μg/mL (QCL), 10 μg/mL (QCM), and 37.5 μg/mL (QCH).

### 2.6 System suitability test

System suitability tests were carried out to ensure that the analytical conditions in the chromatography system met the requirements to obtain precise results. A mixture of phenytoin and carbamazepine standard solution with a concentration of 10 μg/mL each was taken and injected into the HPLC-PDA system for six times. The area and retention time were recorded to find the coefficient of variation (%CV) value from five injections. The requirement was a %CV value of 2% or less (Harmita, 2014). %CV was calculated using the formula displayed in Supplementary Figure S1.

### 2.7 Method validation

Partial validation was executed to implement the analytical method that has been fully validated by Jihan (2023) in the same laboratory. Parameters being evaluated included linearity, within-run accuracy and precision.

The calibration curve consisted of seven different concentrations, which were 0.1 μg/mL; 1 μg/mL; 5 μg/mL; 10 μg/mL; 20 μg/mL; 30 μg/mL; and 50 μg/mL. Calibration curve standard solution was injected into the HPLC-PDA system with an injection volume of 20 μL. The analysis produced an area that could be used to create a calibration curve and determine the linear regression equation (Supplementary Figure S4) to obtain the back calculated calibration standard concentration. The concentration may not exceed ± 15% of the nominal concentration (EMA, 2022), except for Lower Limit of Quantification (LLOQ) which may not exceed ± 20% of the nominal concentration. As much as 75% of the six non-zero concentrations need to meet these requirements. This test was carried out three times, thus, at least 50% of the calibration standard concentrations tested should not exceed ± 15% and ± 20% for the LLOQ. Furthermore, calibration curve parameters should be reported, including the curve’s intercept, slope, and correlation coefficient (r), which can be calculated with formulas in Supplementary Figure S3.

Accuracy and precision tests were carried out with five replicates of LLOQ, QCL, QCM, and QCH. Accuracy was the percentage of difference (%diff) from the nominal concentration while precision was expressed as a coefficient of variation (CV) in percentage. The formula to calculate the percentage of difference was the subtraction of nominal concentration from obtained concentration, which was then divided by the nominal concentration and multiplied by 100 (Supplementary Figure S2). %CV was calculated by dividing standard of deviation with the mean concentration (Supplementary Figure S1). The acceptance criteria for accuracy were not to exceed ± 15% of the nominal concentration and not to exceed ± 20% for LLOQ. Meanwhile, the acceptance criteria for precision were CV not exceeding ± 15% for QCL, QCM, and QCH, and not exceeding ± 20% for LLOQ (US FDA, 2022).

### 2.8 Blood sample collection

Before blood collection, an ethical approval with document number of KET-163/UN2.F1/ETIK/PPM.00.02/2023 was received from the local academic hospital after careful review of the research. Healthy subjects were admitted to the BA/BE Laboratory a day before sampling was conducted. They must undergo a fast for 8 h before being given a 100 mg phenytoin capsule with 240 mL of water. Blood sampling was carried out 15 times starting 30 min before drug administration (pre-dose), and followed by sampling at 0.5, 1, 1.5, 2, 2.5, 3, 4, 6, 9, 12, 24, 36, 48, and 72 h after drug administration. Blood samples were taken as much as 100 μL from the peripheral vessels at the fingertip using a lancet with the finger prick technique. The collection was carried out by a phlebotomist and monitored by a doctor. The blood samples obtained were collected in a tube and 30 μL was spotted on DBS paper with a calibrated micropipette. The DBS paper containing the subject’s blood was dried at room temperature for 120 min and packed into a zip lock bag to be stored in a refrigerator at 2^°^–8^°^C. During blood sampling, food and drink consumed by the subjects must be standardized.

### 2.9 Sample preparation

The blood sample on the DBS paper was cut according to the size of the spot and inserted into the microtube. Then, 30 μL of 10 μg/mL carbamazepine and 400 μL methanol were added to the microtube. The mixture was shaken with a vortex for 30 s, sonicated for 15 min, and centrifuged at 10,000 rpm for 5 min. The centrifuged supernatant was transferred into a new microtube and evaporated with N_2_ gas. The residue obtained then went through a reconstitution process with 100 μL of mobile phase. The residual solution was shaken again with a vortex for 30 s, sonicated for 2 min, and centrifuged at 10,000 rpm for 3 min. This supernatant was transferred to the autosampler vial for analysis.

### 2.10 Pharmacokinetic parameters analysis

Blood samples were analyzed using HPLC-PDA. A comparison of the sample area with the internal standard area was done to obtain a Peak Area Ratio (PAR) value. Phenytoin levels were calculated by interpolating the PAR values to the linear regression equation from the calibration curve obtained (Supplementary Figure S4). The data obtained was plotted into a curve to show the relationship between concentration and time so that pharmacokinetic parameters can be identified, including the maximum concentration of phenytoin in plasma (C_max_), the time required to reach the maximum concentration (t_max_), the half-life (t_1/2_), and the area under curve, namely, AUC_0-t_ and AUC_0-∞_.

All analyses of phenytoin’s pharmacokinetic parameters were performed using the Microsoft Excel software. C_max_ was the highest value among the calculated phenytoin levels and t_max_ was the time associated with C_max_. Half-life was obtained by dividing 0.693 with the rate constant, *k*, which equals to the slope of the curve. The Area Under Curve was attained by calculating the area of a trapezoid between time intervals with the formula shown in Supplementary Figure S5. AUC_0-∞_ could be obtained through the summation of the trapezoidal areas between every pair of consecutive data points, including the last area under the curve that was extrapolated to *t* = ∞.

## 3 Results and discussion

### 3.1 System suitability test

**FIGURE 1:**
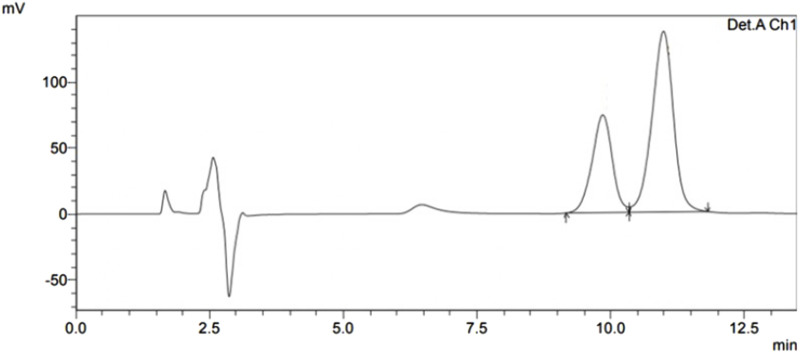
Chromatogram from system suitability test.

The results of the system suitability test in Table 1 showed the average value of phenytoin area was 3,144,813.6 with a CV value of 1.28%, and average carbamazepine area was 3,585,189.4 with a CV value of 1.62%. Meanwhile, the average retention time for phenytoin was 10.174 min with a CV value of 0.45% and the average retention time for carbamazepine was 11.261 min with a CV value of 0.37%. Based on these results, this test was concluded to meet the requirements because the CV values were below 2.0%. The sample chromatogram can be found in Figure 1.

**TABLE 1:** System suitability test data.

No.	Area (μV/s)		Retention time (minutes)	
	Phenytoin	Carbamazepine	Phenytoin	Carbamazepine
1	3,112,653	3,610,476	10.134	11.225
2	3,119,864	3,644,099	10.117	11.209
3	3,137,200	3,517,170	10.203	11.302
4	3,213,930	3,625,489	10.202	11.296
5	3,140,421	3,528,713	10.216	11.272
Mean	3,144,814	3,585,189	10.174	11.261
SD^a^	40,346	58,202	0.05	0.04
CV^b^ (%)	1.28%	1.62%	0.45%	0.37%

^a^ SD, standard deviation, calculated with the method explained in Section 2.6.

^b^ CV, coefficient of variation, calculated with the method explained in Section 2.6.

### 3.2 Method validation

#### 3.2.1 Calibration curve

**FIGURE 2:**
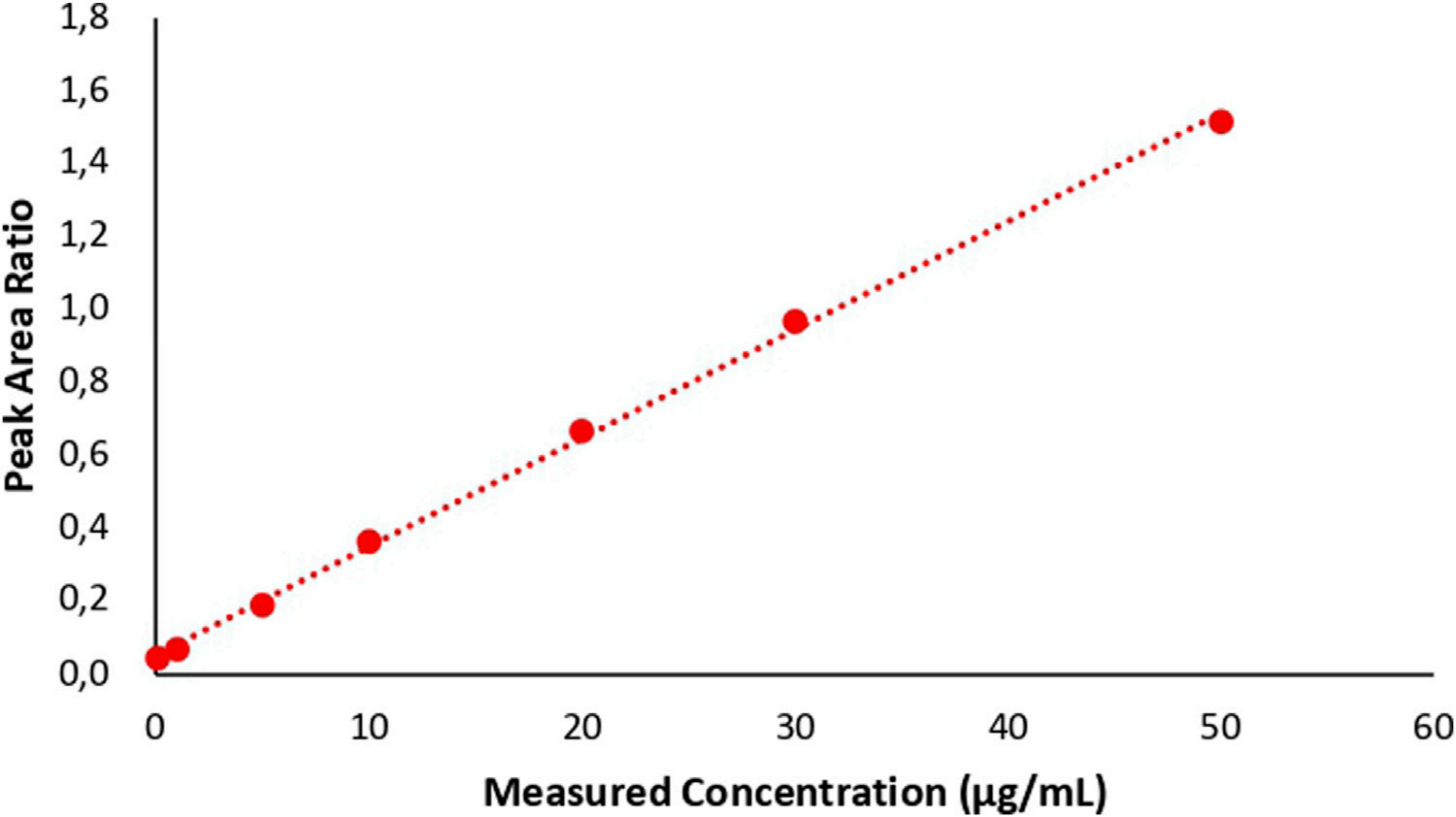
Calibration curve.

Based on these data, the linear regression equation obtained was y = 0.0301x + 0.0405 with x as the concentration of phenytoin (μg/mL) and y as the peak area ratio (PAR) of phenytoin with carbamazepine. The calibration curve can be found in Figure 2 and the data can be found in Table 2. The slope, intercept, and correlation coefficient values obtained were 0.0301, 0.0405, and 0.9990 respectively. The results of calibration curve evaluation met the requirements because the %diff value for LLOQ concentrations did not exceed ± 20% and the %diff for concentrations other than LLOQ did not exceed ± 15%. In addition, the correlation coefficient value fulfilled the linearity requirement because the criteria for r was larger than 0.99 for a biological matrix (Carpenter et al., 2023).

**TABLE 2:** Calibration curve data.

Nominal concen-tration (μg/mL)	Area (μV/s)		PAR^a^	Measured concentration (μg/mL)	*%diff* ^b^
	Phenytoin	Carbamazepine			
0.0	0	1.222.428	0.0000	0	0.00
0.1	35.550	816.311	0.0435	0.1013	1.31
1.0	76.165	1.101.255	0.0692	0.9522	−4.78
5.0	124.096	652.068	0.1903	4,9771	−0.46
10.0	337.333	926.582	0.3641	10,7496	7.50
20.0	480.693	721.146	0.6666	20,7996	4.00
30.0	930.531	962.124	0.9672	30,7862	2.62
50.0	126.4695	834.203	1,5161	49,0217	−1.96

Slope^c^ (a)	Intercept^c^ (b)		r		*r* ^2^
0.0301	0.0405		0.9990		0.9980

^a^ PAR, peak area ratio, calculated with the method explained in section 2.7.

^b^ *%diff* = Percentage of difference, calculated with the method explained in section 2.7.

^c^ Slope, intercept, and r value were calculated with the method explained in section 2.7.

#### 3.2.2 Accuracy and precision

Based on the data which can be found in Table 3, the range of %diff values of the five samples tested at LLOQ concentrations was 0.95%–11.05% with %CV of 4.18%; at QCL concentration was −3.18%–11.63% with %CV of 5.64%; at QCM concentration was 0.43%–13.9% with a %CV of 4.96%; and at QCH concentration was −1.26%–5.59% with a %CV of 2.74%. Evaluation of accuracy and precision met the requirements because all %diff and CV values were below ± 15%.

**TABLE 3:** Within-run accuracy and precision data.

Nominal concentration (μg/mL)	Measured concentration (μg/mL)			
	Mean (μg/mL)	SD^a^	CV^b^ (%)	*%diff* ^c^
Lower Limit of Quantification 0.10	0.11	0.00	4.18	0.95–11.05
Quality Control Low 0.30	0.32	0.02	5.64	−3.18–11.63
Quality Control Medium 10.00	10.96	0.54	4.96	0.43–13.90
Quality Control High 37.50	38.74	1.06	2.74	−1.26–5.59

^a^ SD, standard deviation, calculated with the method explained in section 2.7.

^b^ CV, coefficient of variation, calculated with the method explained in section 2.7.

^c^ *%diff* = Percentage of difference, calculated with the method explained in section 2.7.

### 3.3 Phenytoin pharmacokinetic parameters

**FIGURE 3:**
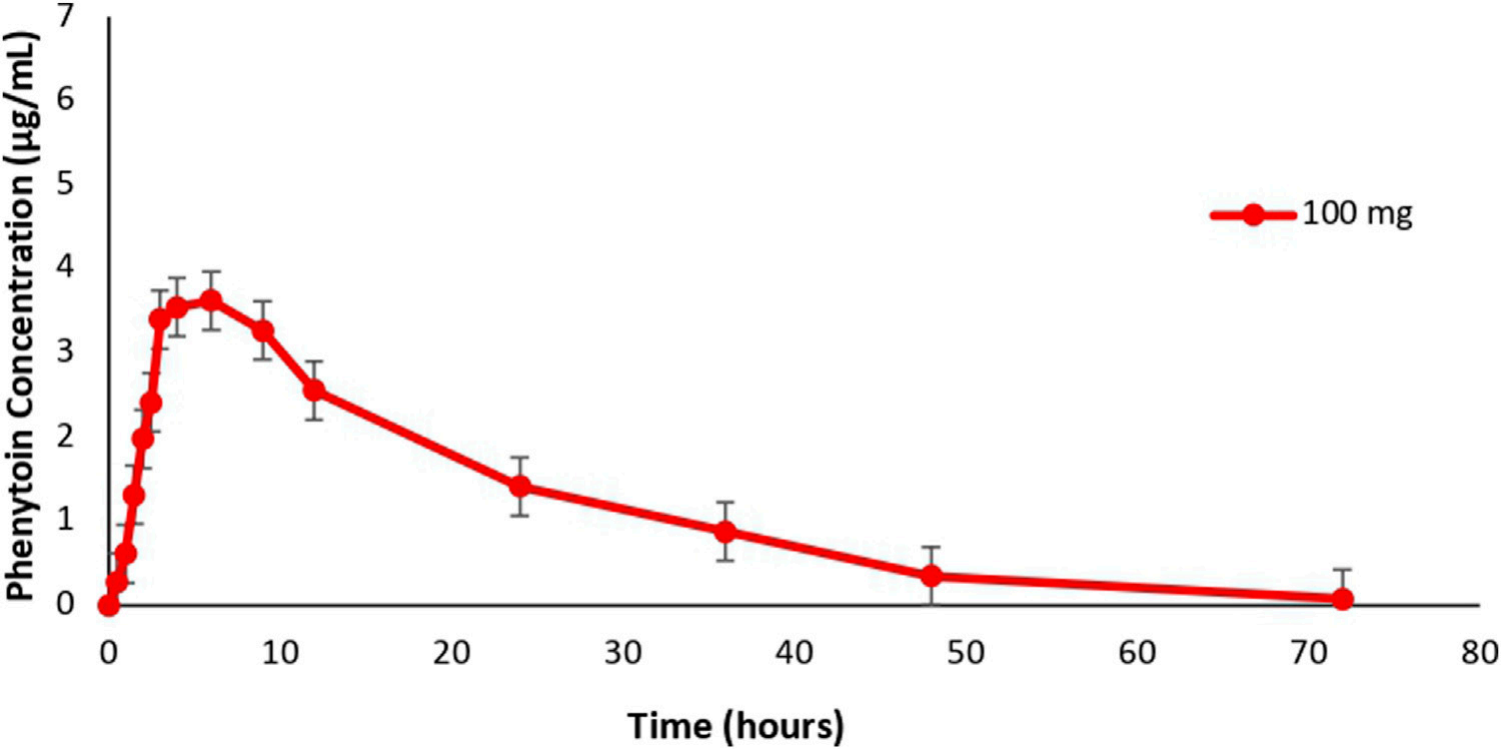
Mean phenytoin concentration-time curve.

Phenytoin pharmacokinetic parameters of 6 subjects obtained from the study can be found in Table 4 and the profile can be found in Figure 3. The maximum concentration of total phenytoin levels obtained was in the range of 3.18 μg/mL to 4.90 μg/mL with an average of 4.33 ± 0.62 μg/mL, while the time needed to reach this concentration was in the range of 3 h–9 h with an average of 6.17 ± 2.48 h. A similar average of t_max_ was obtained by Alegete et al. (2017) who determined the pharmacokinetic profile of phenytoin in plasma, which was 6.67 h after drug administration. Meanwhile, the average C_max_ obtained was higher than the C_max_ value quantified by Rojanasthien et al. (2007) in the plasma matrix after subjects were administered phenytoin 100 mg, which was 2.12 μg/mL. This could be caused by spikes in plasma drug concentrations often associated with antiepileptic drugs, especially with immediate-release dosage form (Leppik and Hovinga, 2013). Comparison of the pharmacokinetic profiles of immediate-release and extended-release phenytoin preparations showed that immediate-release drug products are more bioavailable than extended-release products (Rojanasthien et al., 2007).

**TABLE 4:** Pharmacokinetic parameters of phenytoin in DBS matrix.

No.	C_max_ ^a^ (μg/mL)	t_max_ ^b^ (hours)	t_1/2_ ^c^ (hours)	AUC_0-t_ ^d^ (μg.hrs/mL)	AUC_0-∞_ ^e^ (μg.hrs/mL)	AUC_0-t_/AUC_0-∞_ (%)
SN01	4.51	3.00	8.93	29.15	33.84	86
SN02	4.32	6.00	7.69	90.14	90.85	99
SN03	4.90	9.00	14.17	144.12	150.43	96
SN04	3.18	9.00	10.26	87.88	89.37	98
SN05	4.27	6.00	15.54	84.53	105.91	80
SN06	4.82	4.00	10.39	67.06	67.53	99
**Mean**	4.33	6.17	11.16	83.81	89.65	93
**DS**	0.62	2.48	3.05	37.32	38.89	0.08
**CV (%)**	14%	40%	27%	45%	43%	9%

^a^ C_max_ = Maximum concentration, calculated with the method explained in section 2.10.

^b^ t_max_ = Time for maximum concentration, calculated with the method explained in section 2.10.

^c^ t_1/2_ = Half-life, calculated with the method explained in section 2.10.

^d^ AUC_0-t_ = Area under curve from time zero to time of last observed concentration, calculated with the method explained in section 2.10.

^e^ AUC_0-∞_ = area under curve from time zero to infinity, calculated with the method explained in section 2.10.

^f^ SD, standard deviation, calculated with the method explained in section 2.7.

^g^ CV, coefficient of variation, calculated with the method explained in section 2.7.

The half-life of phenytoin (t_1/2_) obtained from this study was in the range of 7.69 h–15.54 h with an average of 11.16 ± 3.05 h. The value was not different from the study of Randinitis et al. (1990) and Rojanasthien et al. (2007), who determined the phenytoin profile in the plasma matrix and found the half-life value of phenytoin to be 11.3 h and 12.65 h, respectively. Meanwhile, the AUC_0-t_ parameters of the six subjects ranged from 29.15 μg.h/mL to 144.12 μg.h/mL with an average of 83.81 ± 37.32 μg.h/mL and the AUC_0-∞_ was in the range of 33.84 µg.h/mL to 150.43 µg.ghour/mL with an average of 83.65 ± 38.89 µg.h/mL. Both values were different from previous studies. Dalmora et al. (2009) who quantified phenytoin in the plasma matrix found an average AUC_0-t_ of 39.41 ± 8.57 μg.h/mL and an average AUC_0-∞_ of 42.94 ± 9.55 μg.h/mL. This value was also quite different from that of Rojanasthien et al. (2007) which showed an average AUC_0-∞_ phenytoin of 50.92 µg.h/mL. However, the pharmacokinetic profile of subjects produced by plotting data points to the mean phenytoin concentration-time curve was similar to the curves displayed in previous studies conducted in plasma matrix. Direct comparison of concentration curves was done in addition to calculating the pharmacokinetic parameters for a better understanding of the drug’s pharmacokinetics because plasma concentration curve had been used for bioavailability and bioequivalence study (Liao, 2005). Based on these results, the conclusion obtained was that although there were differences between the C_max_ and AUC parameters of phenytoin from the DBS matrix and the plasma matrix, similar t_max_ and half-life were obtained from the two matrices as well as similar pharmacokinetic profiles as indicated by the comparable concentration-time curves.

Variability of pharmacokinetic parameters between individuals could be explained by differences in response to phenytoin which was influenced by the presence of genetic polymorphism in the CYP2C9 enzyme, an enzyme that plays a role in phenytoin metabolism. Previous research showed an increase in phenytoin concentrations in epilepsy patients with the CYP2C9*2 and CYP2C9*3 alleles compared to patients who had the wild-type gene (Silvado et al., 2018). Individuals with one or two copies of the *2 and *3 variants have AUC values that are 4–5 times higher with lower metabolic and clearance rates compared to normal metabolizers (Chang et al., 2020). The frequency of the CYP2C9*3 allele was found to be highest in Asian populations, reaching 11.9% in Pakistan and 11.6% in Bangladesh (Zhou et al., 2023). Therefore, in phenytoin therapy, the CYP2C9 genotype is a factor that can influence the pharmacokinetics of phenytoin.

## 4 Conclusion

Despite differences between the pharmacokinetic parameters of phenytoin analyzed with Dried Blood Spot and plasma matrices, the pharmacokinetic profiles obtained were similar. Hence, the DBS assay can be used interchangeably with plasma assay to quantify total phenytoin concentration in patients receiving phenytoin as therapy.

## Data Availability

The original contributions presented in the study are included in the article/Supplementary Material, further inquiries can be directed to the corresponding author.
