# Supplementary material for: Pharmacokinetic profile of phenytoin in dried blood spot with high-performance liquid chromatography—photodiode array
Source: Front Pharmacol. 2024 Jun 26;15:1326996. doi: 10.3389/fphar.2024.1326996 (PMC11233438; doi:10.3389/fphar.2024.1326996)
Supplement: Supplementary file 1 [file DataSheet1.docx]

Supplementary Material

Pharmacokinetic profile of phenytoin in dried blood spot with high-performance liquid chromatography – photodiode array

**Yahdiana Harahap^1,2^, Limeylia Ng^1*^, Sunarsih^3^**

*** Correspondence: Limeylia Ng**Corresponding Author
limeyliang78@gmail.com

## Supplementary Figures


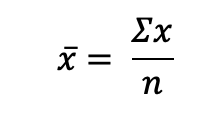

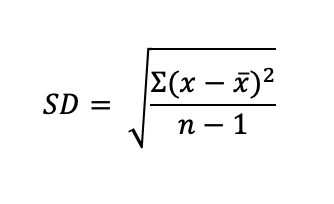

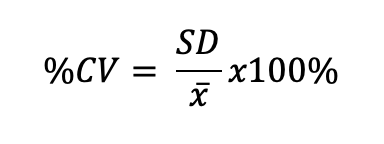


**Supplementary Figure 1.** Mean, standard deviation, and %CV formula where *x̅* = mean value, SD = standard deviation, *%CV* = coefficient of variation*, x* = individual sample value, and *n* = total number of samples.


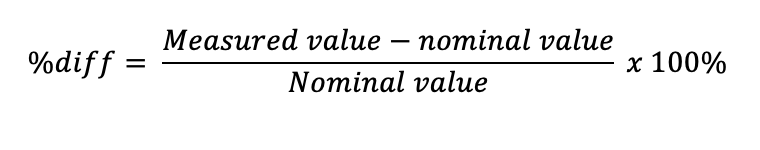


**Supplementary Figure 2.** *%diff* formula where *%diff* = percentage of difference between the measured and nominal values.


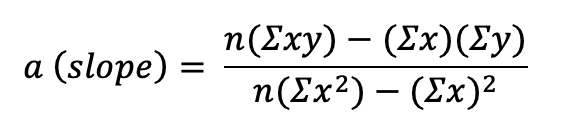

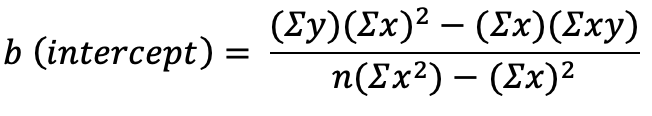

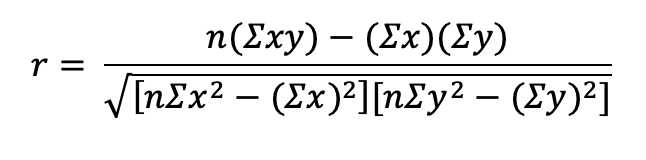


**Supplementary Figure 3.** Calibration curve parameters calculation, including slope, intercept, and coefficient correlation, where *x* and *y* = data points on the curve.


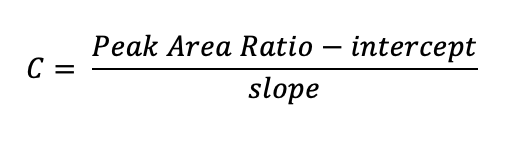


**Supplementary Figure 4.** Concentration calculation using linear regression equation where Peak Area Ratio = obtained area of phenytoin divided by carbamazepine.


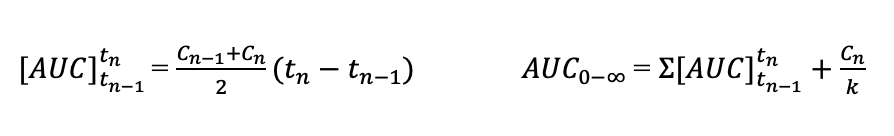


**Supplementary Figure 5.** Area under curve calculation formula where [AUC] = area under the curve, *t_n_* = time of observation of drug concentration *C_n_*, *t_n–1_* = time of prior observation of drug concentration corresponding to *C_n–1_*, *C_n_* = last observed concentration at *t_n_*, and *k* = slope from the curve.
